# Supplementary material for: Transmission of Xylella fastidiosa Subspecies Pauca Sequence Type 53 by Different Insect Species
Source: Insects. 2019 Sep 29;10(10):324. doi: 10.3390/insects10100324 (PMC6835679; doi:10.3390/insects10100324)
Supplement: Supplementary file 1 [file insects-10-00324-s001.pdf]

Supplementary Table S1: Population density of *Philaenus spumarius* and *Neophilaenus. campestris* estimated in 2016 by monitoring on weekly basis six different orchards in the infected area.

Insect species	Vegetational compartment	Period							
		2016							
		April	May	June	July	August	September	October	Monthly average
		Mean (±ES)							
P. spumarius	Olive trees	1.1 ±0.1 b	4.5 ±0.4 a	1.2 ±0.1 b	1.0 ±0.1 b	1.0 ±0.1 ab	0.8 ±0.1 a	0.3 ±0.0 ab	9.8 ±0.1 b
N. campestris		0.1 ±0.0 c	1.0 ±0.3 b	0.0 ±0.0 c	0.0 ±0.0 c	0.0 ±0.0 c	0.0 ±0.0 c	0.1 ±0.0 b	1.2 ±0.04 d
P. spumarius	Ground vegetation	3.0 ±0.3 a	4.4 ±0.4 a	0.2 ±0.0 c	0.0 ±0.0 c	0.3 ±0.0 c	0.4 ±0.1 bc	0.4 ±0.0 a	8.7 ±0.07 b
N. campestris		0.2 ±0.1 c	0.6 ±0.2 b	0.0 ±0.0 c	0.0 ±0.0 c	0.0 ±0.0 c	0.0 ±0.0 c	0.1 ±0.0 b	0.9 ±0.01 d
P. spumarius	Border plants	0.9 ±0.2 b	5.4 ±0.5 a	3.3 ±0.4 a	2.4 ±0.2 a	1.1 ±0.1 a	0.7 ±0.1 ab	0.3 ±0.1 ab	14.0 ±0.2 a
N. campestris		0.1 ±0.1 c	1.0 ±0.2 b	1.4 ±0.1 b	1.1 ±0.1 b	0.7 ±0.1 b	0.7 ±0.1 a	0.4 ±0.1 a	5.2 ±0.06 c

	April	May	June	July	August	September	October	Monthly average
**F value**	46.19	28.89	44.17	81.94	28.74	16.24	4.4	57.28
**Pr > F**	<0.0001	<0.0001	<0.0001	<0.0001	<0.0001	<0.0001	0.0024	<0.0001
**d**	5	5	5	5	5	5	5	5
**Significance**	***	***	***	***	***	***	**	***

Supplementary Table S2: Population density of *Philaenus spumarius* and *Neophilaenus. campestris* estimated in 2017 by monitoring on weekly basis six different orchards in the infected area.

Insect species	Vegetational compartment	Period							
		2017							
		April	May	June	July	August	September	October	Monthly average
		Mean (±ES)							
P. spumarius	Olive trees	0.1 ±0.0 b	2.6 ±0.2 b	0.3 ±0.1 c	0.4 ±0.1 b	0.4 ±0.1 b	0.4 ±0.1 ab	0.1 ±0.0 b	4.2 ±0.07 b
N. campestris		0.1 ±0.0 b	0.8 ±0.1 d	0.1 ±0.0 c	0.0 ±0.0 b	0.1 ±0.0 b	0.1 ±0.0 b	0.1 ±0.0 b	1.1 ±0.03 d
P. spumarius	Ground vegetation	0.7 ±0.0 a	5.4 ±0.1 cd	0.0 ±0.0 c	0.0 ±0.0 b	0.1 ±0.0 b	0.0 ±0.0 b	1.3 ±0.0 a	7.4 ±0.03 c
N. campestris		0.8 ±0.1 a	3.0 ±0.1 d	0.0 ±0.0 c	0.0 ±0.0 b	0.0 ±0.0 b	0.0 ±0.0 b	1.1 ±0.0 a	4.9 ±0.02 cd
P. spumarius	Border plants	0.0 ±0.0 b	6.7 ±0.4 a	2.2 ±0.2 a	1.2 ±0.2 a	0.9 ±0.2 a	0.7 ±0.1 a	0.1 ±0.0 b	11.8 ±0.4 a
N. campestris		0.1 ±0.0 b	2.1 ±0.2 bc	1.1 ±0.1 b	0.2 ±0.1 b	0.4 ±0.1 b	0.4 ±0.1 a	0.2 ±0.0 b	4.5 ±0.1 b

	April	May	June	July	August	September	October	Monthly average
**F value**	23.94	105.00	73.37	13.45	10.18	7.04	68.48	244.73
**Pr > F**	<0.0001	<0.0001	<0.0001	<0.0001	<0.0001	0.0001	<0.0001	<0.0001
**d**	5	5	5	5	5	5	5	5
**Significance**	***	***	***	***	***	***	***	***

Supplementary Table S3: Results of the real time PCR reactions performed in 2016 and 2017 on the insect specimens collected in infected olive groves throughout the spittlebug adult season.

Insect species	Vegetational compartment	2016											
		May		June		July		August		September		October	
		No. of insects		No. of insects		No. of insects		No. of insects		No. of insects		No. of insects	
		Tested	Positive (%)	Tested	Positive (%)	Tested	Positive (%)	Tested	Positive (%)	Tested	Positive (%)	Tested	Positive (%)
Philaenus spumarius	Olive trees	200	21 (10.5)	199	101 (50.75)	200	99 (49.5)	194	87 (44.84)	157	86 (54.78)	58	29 (50)
Neophilaenus campestris		75	0	7	0	0	0	2	0	9	0	19	2 (10.53)
P. spumarius	Ground vegetation	80	3 (3.75)	0	0	0	0	40	15 (37.5)	21	5 (23.81)	60	35 (58.33)
N. campestris		71	2 (2.82)	0	0	1	0	2	0	9	0	31	0
P. spumarius	Border plants	100	6 (6)	120	13 (10.83)	40	8 (20)	54	20 (37.04)	40	12 (30)	13	6 (46.15)
N. campestris		15	0	4	0	1	0	41	0	49	0	24	1 (4.17)
2017													
P. spumarius	Olive trees	160	16 (10)	44	22 (50)	59	17 (28.81)	40	7 (17.5)	39	8 (20.51)	0	0
N. campestris		94	7 (7.45)	0	0	0	0	0	0	7	0	33	0
P. spumarius	Ground vegetation	80	2 (2.5)	0	0	0	0	0	0	38	7 (18.42)	0	0
N. campestris		40	0	0	0	0	0	0	0	1	0	32	0
P. spumarius	Border plants	160	7 (4.37)	80	9 (11.25)	20	1 (5)	39	5 (12.82)	39	9 (23.07)	6	0
N. campestris		118	1 (0.85)	44	0	5	0	10	0	14	0	14	0

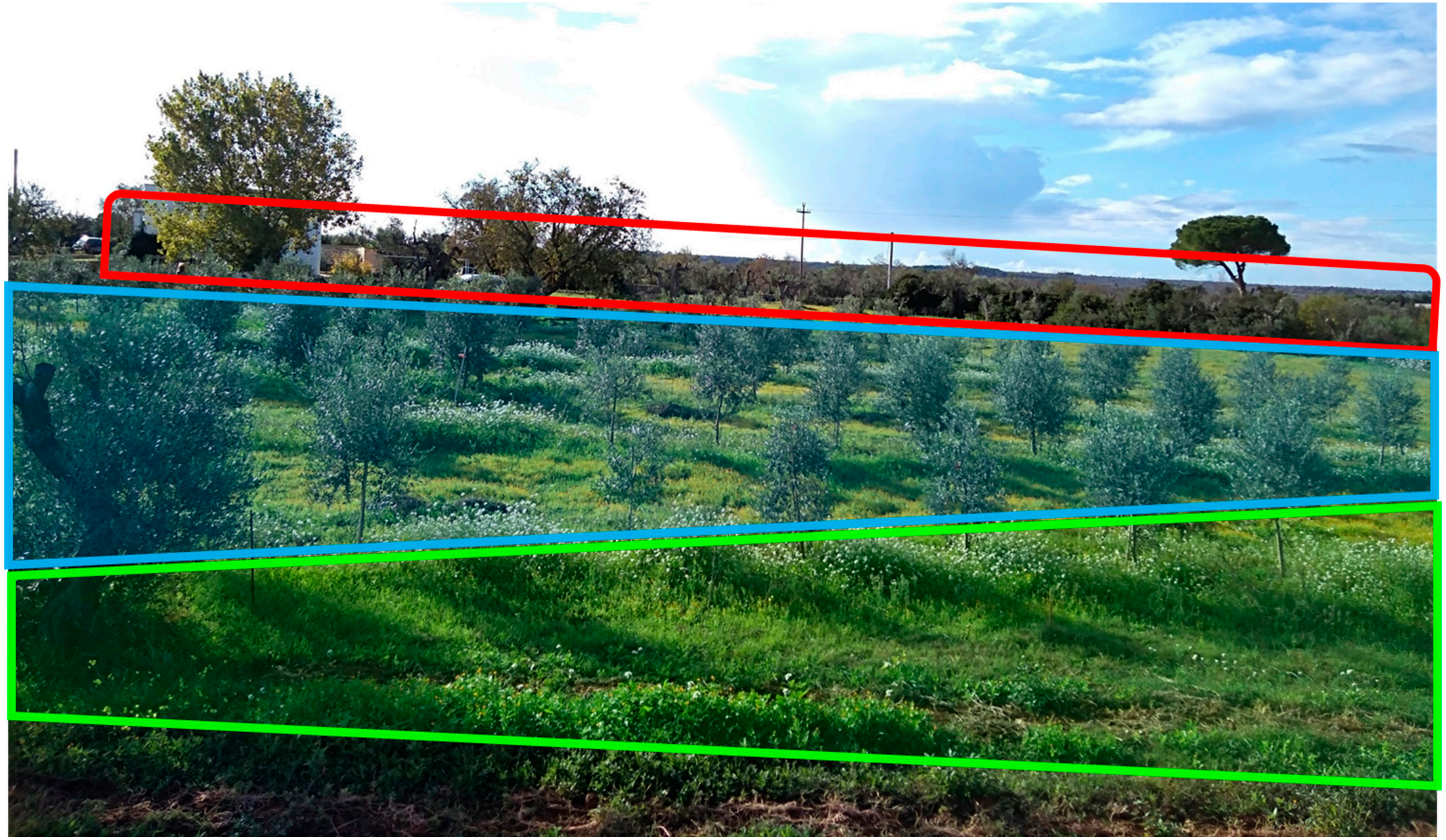

Supplementary Figure S1: Olive orchard selected in the demarcated infected area of Apulia region (southern Italy) for monitoring and collecting spittlebugs in different vegetation compartments:olive canopies (blue rectangle), ground cover vegetation (green rectangle) and border trees/shrubs (red rectangle).

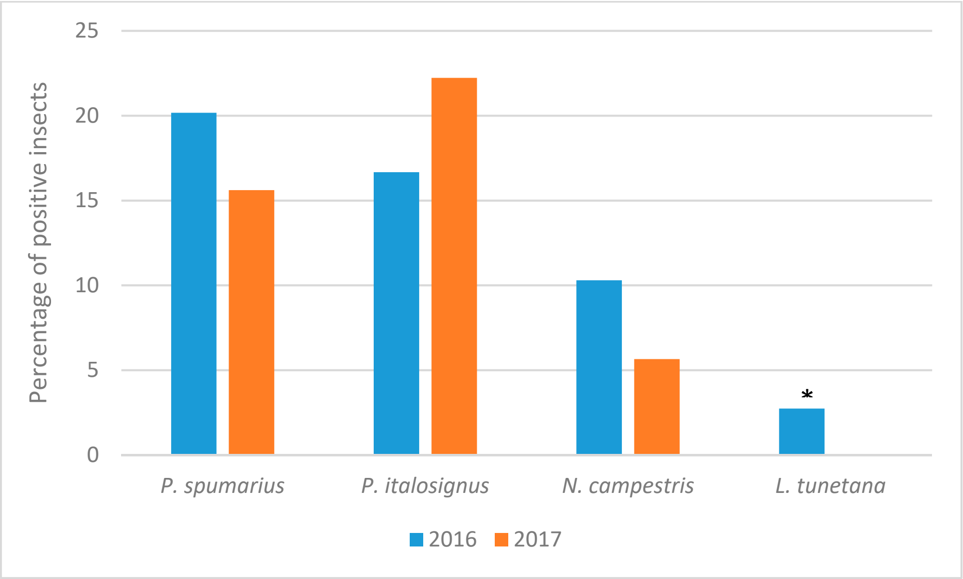

Supplementary Figure S2. Percentage of insects testing positive in real time PCR in 2016 and 2017, after 96 h of acquisition access period, for *Philaenus spumarius*, *P. italosignus* and *Neophilaenus campestris. *Adults of Latilica tunetana were collected directly from the canopies of field-infected trees*
